# A convenient viral transduction based method for advanced multi-engineering of primary human (CAR) T-cells

**DOI:** 10.1016/j.jgeb.2024.100446

**Published:** 2024-11-28

**Authors:** Jort J van der Schans, Afroditi Katsarou, George Kladis, Citlali Bar, Max Medina Ramirez, Maria Themeli, Tuna Mutis

**Affiliations:** Department of Hematology, Amsterdam UMC, Location VU University Medical Center, Cancer Center Amsterdam, Room CCA3.38, De Boelelaan 1117, Amsterdam 1081 HV, the Netherlands

**Keywords:** Y-retrovirus, T-cell engineering, Stable and simultaneous virus production, Double, Triple transduction

## Abstract

The past decades have illustrated the power of T-cell engineering in the development of new and successful cell therapies, such as chimeric antigen receptor (CAR) T-cells. Despite clinical success in hematological malignancies, it also becomes increasingly clear that additional T-cell engineering will be required to improve efficacy and safety and expand the application to solid tumors. Engineering is most often achieved by viral delivery of transgenes, however, viral vector capacity limitations make efficient and reproducible generation of multi transgene expressing T-cell therapeutics technically challenging. We here describe a convenient and efficient method for the delivery of up to three γ-retroviral CAR vectors in T-cells. We achieved this using virus vector mixtures that are simultaneously produced at high titers by double- or triple- transduced stable virus producer cells. We show that this method is superior in overall efficiency and reproducibility to conventional double or triple CAR transductions, in which separate viral batches are used. Due to its robustness, this method can facilitate the research and the development for advanced T-cell engineering towards more effective and safe therapies.

## 1 Introduction

Over the past decades, genetically engineered T-cells and especially T-cells expressing tumor-reactive chimeric antigen receptors (CAR T-cells), have emerged as a promising and effective treatment modality for various hematological malignancies.^1^, ^2^, ^3^ While the initial responses to most CAR T-cell therapies are impressive, a considerable proportion of patients relapses. Tumor escape due to loss of target expression, poor CAR T-cell persistence and the immunosuppressive tumor microenvironment, especially in solid tumors, have currently been identified as the major challenges towards a more effective and safe CAR T-cell therapy.^4^, ^5^, ^6^

Solutions to overcome these challenges may be found in the advanced engineering of T-cells, which could involve the incorporation of additional (synthetic) receptors or ligands (e.g. CARs, chimeric costimulatory receptors, synthetic Notch receptors, 4-1BB ligand) to counter antigen-loss-related tumor evasion,^7^, ^8^, ^9^, ^10^, ^11^ the introduction of stimulatory or pro-inflammatory cytokine genes to enhance their durability and/or modulate the immunosuppressive tumor microenvironment^9,^^10^ or the integration of safety switches and performance-enhancing genes to enhance safety, specificity, or metabolic profile.^12^, ^13^, ^14^, ^15^, ^16^ Nevertheless generating these next-generation CAR T-cells, and especially combining these novel beneficial strategies poses a great challenge, as this requires the successful incorporation and expression of multiple transgenes within the same T-cell. Stable transgene expression in a T-cell can in principle be achieved through viral and non-viral gene delivery. Examples of non-viral delivery are transposon-mediated gene delivery and knock-in using genome editing tools (e.g. CRISPR/Cas9, PRIME editing).^17^, ^18^, ^19^ Although both approaches are upcoming and promising, so far they result in lower efficacy compared to well-established viral gene delivery methods in T-cells.^20^ Hence, viral gene delivery, mostly by lentivirus or γ-retrovirus, remains the preferred approach for the delivery and stable expression of genes of interest in human T-cells.

Expressing multiple transgenes in T-cells via viral gene transfer can be accomplished using either bi-, tri-, or multi-cistronic vectors^21^ or by employing two or more vectors, which may be polycistronic on their own. While the former strategy offers the advantage of a single insertion into the host genome, the limited cargo capacity of lenti- and γ-retroviral vectors and the often inefficient expression of downstream open-reading frames are significant disadvantages of using polycistronic vectors.^22^, ^23^ The drawback of employing double or triple transductions is the generation of mixed populations of T cells expressing single, double or triple transgenes.^21^ Frequently, the proportion of the desired double or triple transduced T-cells is relatively low, presenting a technical challenge to obtain sufficient numbers of desired cells even after intensive cell sorting.

To overcome these difficulties we now employed a straightforward yet effective method for achieving high levels of double and triple CAR-transduced T-cells. To this end, we used the 293vec-RD114 virus producer cell line,^24^, ^25^ which is pseudotyped to efficiently infect human lymphocytes and hematopoietic stem cells. We simultaneously transduced them with up to three γ-retroviral vectors encoding three different CAR transgenes. The successfully transduced producer cells were sorted to establish “master” cell lines that can simultaneously and stably produce all retroviral vectors in one mix. We then tested the efficiency and reproducibility of this viral vector-mix to generate functional triple-transduced CAR T-cells.

## 2 Materials & methods

### 2.1 Primary cells from MM patients and healthy individuals

Peripheral blood mononuclear cells (PBMCs) were isolated from buffy coats of healthy blood bank donors by Ficoll-Paque density centrifugation. All patient material was collected after written informed consent was obtained according to the code of conduct for medical research developed by The Council of the Federation of Medical Scientific Societies (FEDERA, https://www.coreon.org/wp-content/uploads/2023/06/Code-of-Conduct-for-Health-Research-2022.pdf). All procedures were performed in accordance with the Declaration of Helsinki.

### 2.2 Cell lines

293vec-RD114 cells were provided by Dr. M. Caruso (CHU de Québec-Université Laval Research Center (Oncology Division)). Virus-producer cell lines Phoenix-Ampho (ATCC) and 293vec-RD114 cells were cultured in DMEM + 10 % FCS + antibiotics (penicillin 100U/ml, streptomycin 100ug/ml). The multiple myeloma cell line UM9, and all variants thereof, were authenticated by STR and cultured in RPMI1640 + 10 % FCS + antibiotics (penicillin 100U/ml, streptomycin 100ug/ml). CD38 knockout (CD38KO) was achieved through CRISPR/Cas9 mediated gene disruption as previously described.^12^ UM9-CD38KO overexpressing TIM3 or CLEC12A (UM9-CD38KO-TIM3 and UM9-CD38KO-CLEC12A) were generated through lentiviral transduction of the cells with the pLM vector containing the corresponding gene (NM_032782.5, NM_138337.6). After transduction, surface antigen expression was assessed using flow cytometry. Positive cells were sorted by FACS to a purity of > 95 % and cultured as the untransduced parental cells.

### 2.3 Retroviral constructs

**Fig. 1:**
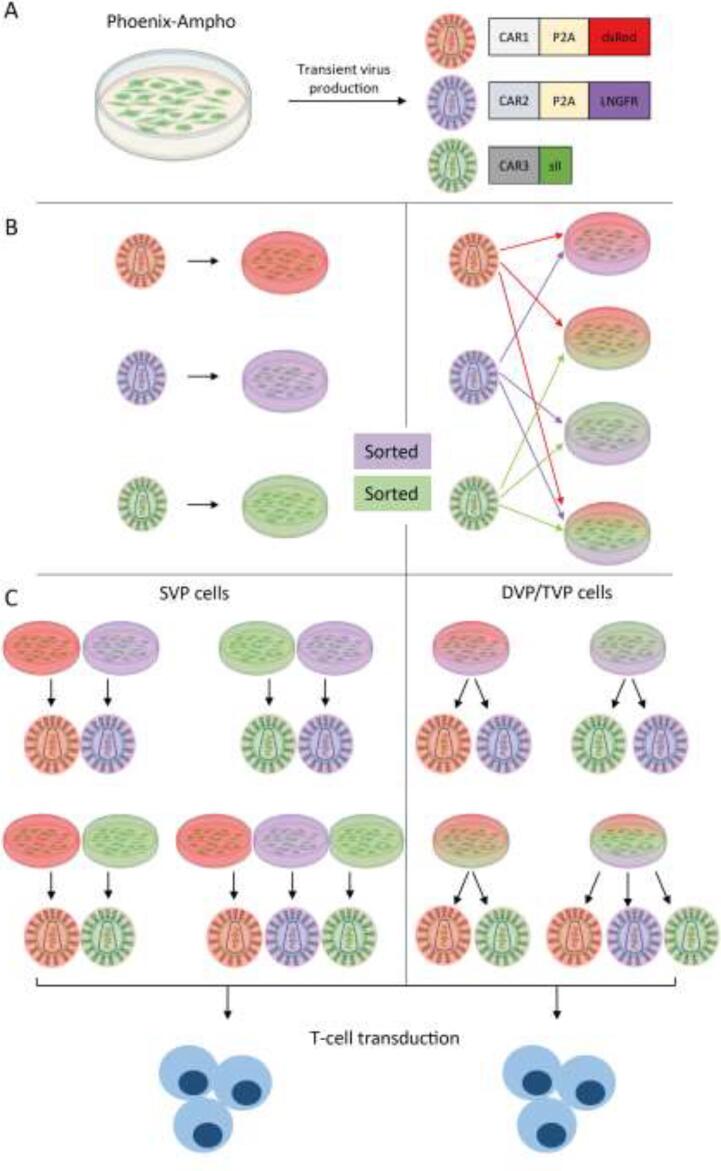
Schematic overview of using single, double or triple virus producer cell derived virus to generate double or triple CAR expressing primary human T-cells. (A) Phoenix-Ampho cells are used to produce CAR virus 1, 2 or 3, which are each labeled with a distinctive marker. (B) 293vec-RD114 cells are single (CAR1, CAR2 or CAR3), double (CAR1 + CAR2, CAR1 + CAR3 or CAR2 + CAR3) or triple transduced (CAR1 + CAR2 + CAR3) with Phoenix-Ampho cell derived virus to generate single, double or triple virus producer cells (SVP, DVP, TVP, respectively). Subsequently VP cells were further purified by positive selection for CAR2 and CAR3 expression. (C) Double and triple CAR transduction of primary human T-cells with DVP and TVP cell derived virus is compared to transduction with mixtures of SVP derived virus as control.

**Fig. 2:**
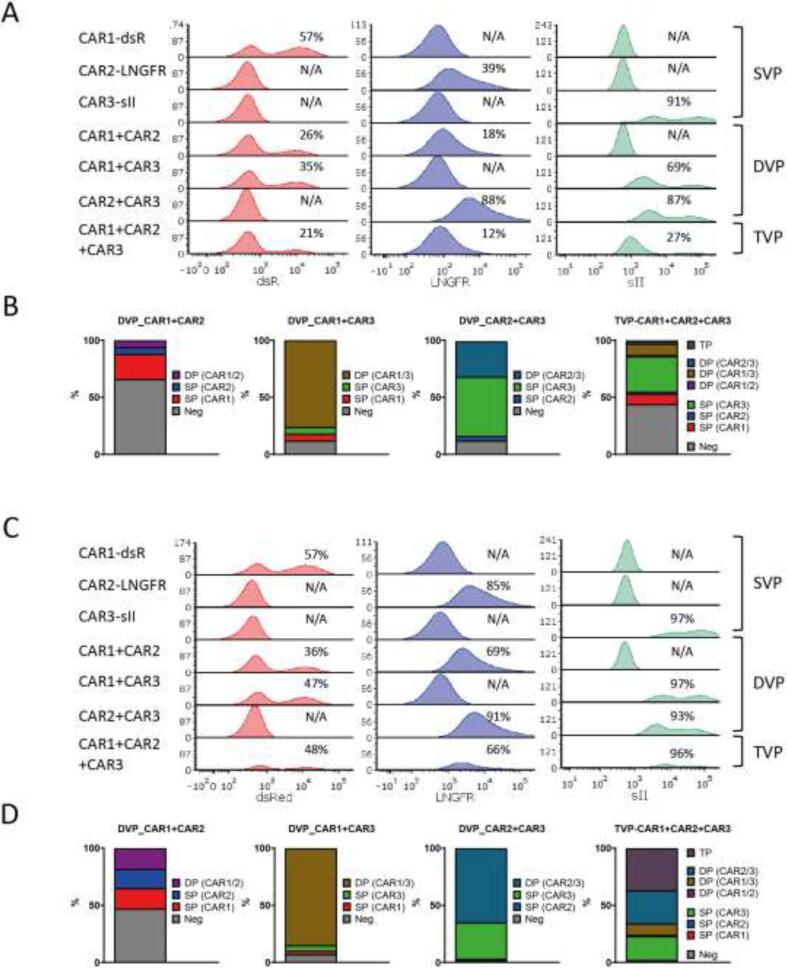
293vec-RD114 cell transduction efficiencies. (A) Representative plots of pre-sort transduction efficiency of CAR1/2/3 in 293vec-RD114 SVP, DVP and TVP cells showing the median fluorescent intensity (MFI) of each CAR marker and (B) accompanied proportions of double and triple positive populations in DVP and TVP cells. (C) Representative plots of post-sort transduction efficiency of CAR1/2/3 in 293vec-RD114 SVP, DVP and TVP cells showing the MFI of each CAR marker and (D) accompanied proportions of double and triple positive populations in DVP and TVP cells. Neg = CAR/marker negative, SP = single CAR/marker positive, DP = double CAR/marker positive, TP = triple CAR/marker positive.

**Fig. 3:**
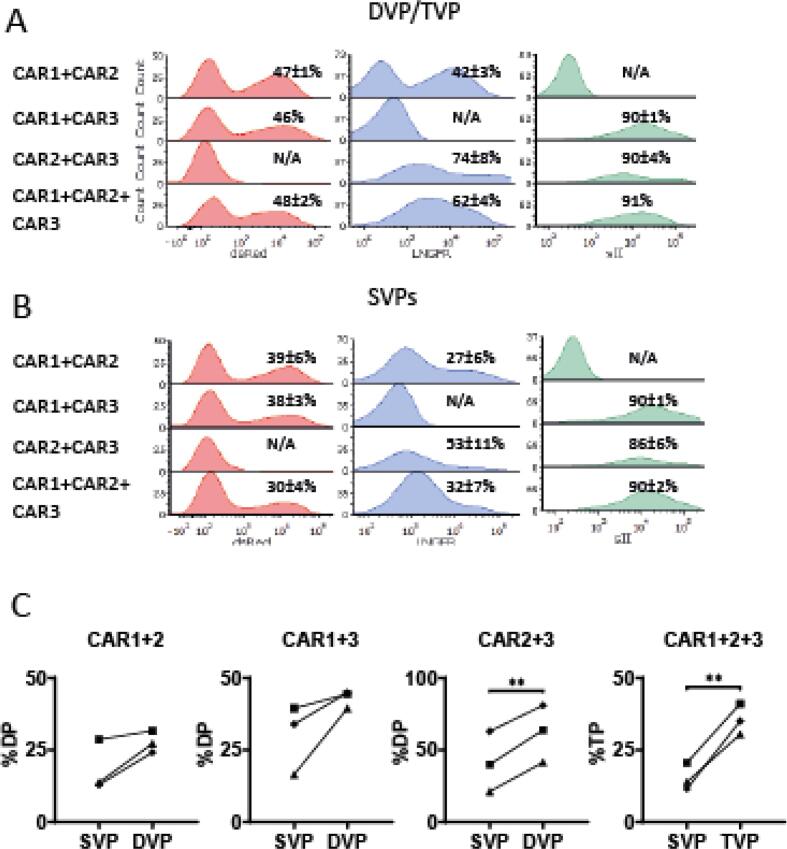
Double and triple CAR T-cell transduction performed with DVP or TVP derived virus. Representative CAR expression histograms showing the median fluorescent intensity of each individual CAR marker in primary human T-cells after double and triple CAR transductions in three donors achieved with (A) DVP or TVP derived virus compared to (B) SVP control. (C) Proportions of double or triple CAR-transduced T-cells achieved with DVP or TVP derived virus compared to SVP control in three T-cell donors. Experiments were performed in duplicates and different symbols represent different T-cell donors. Statistical analysis in (C) was performed by standard paired *t* test of percentages. ** indicates p value < 0.01. SVP = single virus producer cells, DVP = double virus producer cells, TVP = triple virus producer cells, TP = triple positive, DP = double positive, SP = single positive, Neg = negative.

**Fig. 4:**
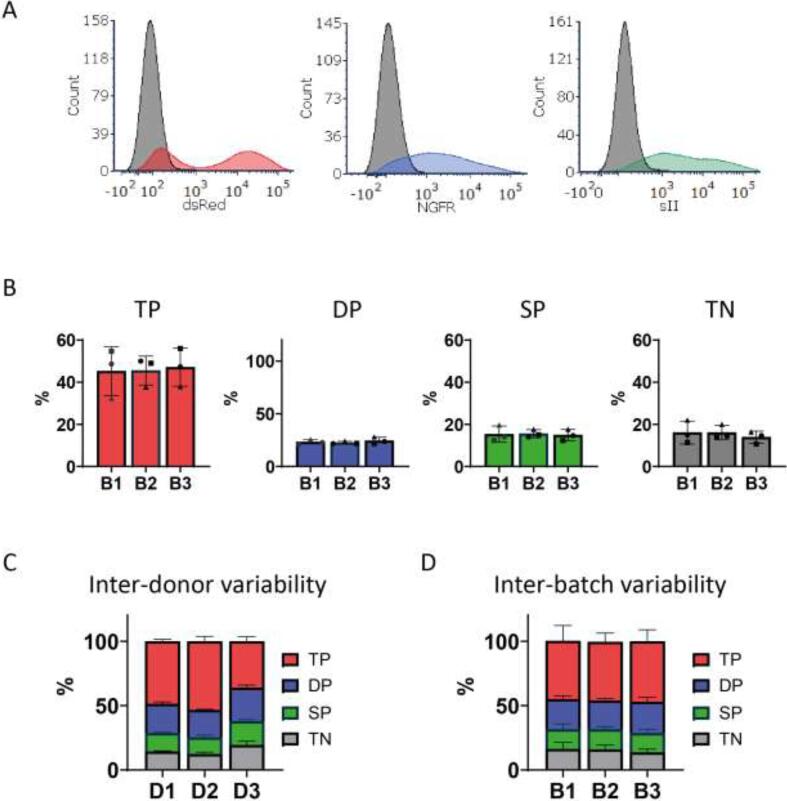
Reproducibility of triple CAR T-cell transduction achieved with TVP derived virus. (A) Representative histograms showing the median fluorescent intensity of CAR marker expression in anti-CD38 + CLEC12A + TIM3 (38/C/T) CAR T-cells and control untransduced T-cells in gray. (B) Percentages of triple CAR positive (TP), double CAR positive (DP), single CAR positive (SP) or triple negative (TN) T-cells from three distinct donors transduced with three independent virus batches (B1-3). (C, D) Variability in 38/C/T CAR T-cell population composition after transduction between healthy primary T-cell donors (D1-3) or virus batches (B1-3). In all experiments *n* = 3 and duplicates, error bars represent mean ± SD. In (B) different symbols each represent a different T-cells donor. In this figure, CAR1 (dsRed) is a previously described high affinity (A1) second generation anti-CD38 CAR with a CD28 costimulatory domain,^29^ transmembrane domain and hinge, CAR2 (LNGFR) is an anti-TIM3 CAR and CAR3 (sII) is an anti-CLEC12a CAR, both containing a CD8a hinge and transmembrane domain and 4-1BB costimulatory domain.

**Fig. 5:**
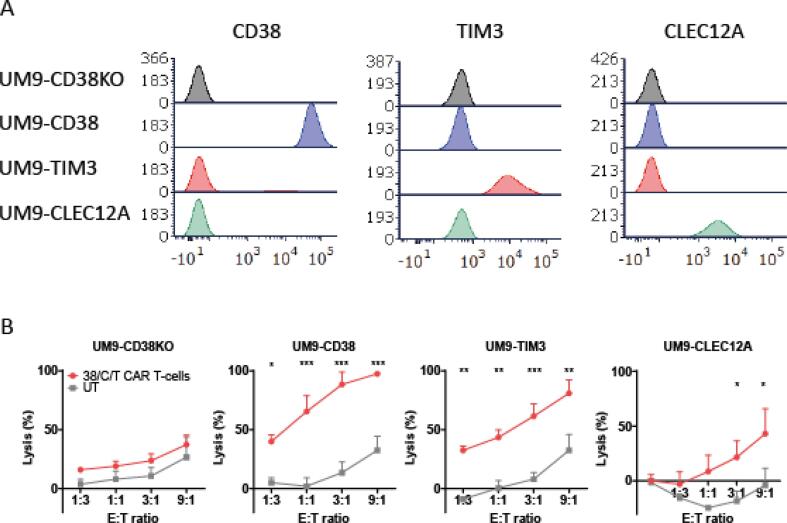
Functional activity of through TVP-virus generated anti-CD38 + TIM3 + CLEC12A CAR T-cells. (A) Histograms showing the median fluorescent intensity of single target positive UM9 cells, expressing no target (UM9-CD38KO) or either CD38, TIM3 or CLEC12A only (UM9-CD38, UM9-TIM3, UM9-CLEC12A, respectively). (B) The cytotoxic activity of anti-CD38 + TIM3 + CLEC12A (38/C/T) CAR T-cells against UM9 cells that express none or only one of the putative CAR target antigens. CAR T-cells or untransduced (UT) T-cells were incubated with target cells in different effector to target (E:T) ratios for 24 h. In all experiments *n* = 3 and duplicates, error bars represent mean ± SD. Statistical analysis in (B) was performed by two-way analysis of variance (ANOVA) and subsequent multiple comparisons. ns = not significant, * indicates p value < 0.05, ** <0.01 and *** <0.001.

All CAR genes used in this study were constructed into the SFG retroviral vector.^26^ Several first or second generation CARs have been used as indicated in the figure legends. For the sake of clarity, in each experimental setting, the different CARs were designated as CAR1, CAR2 and CAR3, although they are not always the same CARs. To be able to detect CAR construct integration in virus producer cells and T-cells and to make sorting of CAR^+^ cells possible, different selection genes were used per CAR construct, indicated in Fig. 1. In general the CAR1 construct contained always the dsRed gene, linked to the CAR sequence through a P2A sequence. The CAR2 construct contained a LNGFR gene as a marker. The CAR3 construct was detected with a strep tag II (sII, NWSHPWFEK), a tag that is commonly used by us and others to detect expression of CARs or other synthetic surface molecules incorporated in between the CAR hinge and transmembrane domain.^12^, ^27^, ^28^ The number of base pairs in between LTRs were 3646 bp, 3730 bp and 2914 bp in Fig. 2, Fig. 3 and 3764 bp, 3940 bp, and 2911 bp in Fig. 4, Fig. 5, for CAR1, CAR2 and CAR3, respectively.

### 2.4 Individual CAR vector production in phoenix-ampho cells

To produce individual CAR vectors, 15ug construct DNA was first transfected into Phoenix-ampho cells using CaCl2 and HEPES-buffered saline, pH 7.0 (Thermo Scientific) in a standard calcium-phosphate technique, as described elsewhere. The next day, supernatants containing viral vectors were harvested, concentrated using Retro-X (TakaraBio), and replaced with 10 ml fresh medium to repeat harvesting and concentration the next day. Concentrated virus from two batches was combined and frozen at −80C until use.

### 2.5 Generation of stable single, double or triple virus producer cell lines and virus harvest

An overview of all 293vec-RD114 cell transductions using Phoenix-Ampho-derived virus is summarized in Table 1. Briefly, one day before transduction, 293vec-RD114 cells (0,3x10^6^ per well) were plated in a gelatin-coated 6-well plate. To transduce the cells, all medium was removed and the cells were incubated with a total volume of 1 ml of concentrated (Phoenix-Ampho-derived) γ-retroviral CAR1, CAR2, CAR3 vectors or a mixture of CAR vectors, as indicated in the results section. After adding 4ul of polybrene, the cells were spinoculated for 1 h at 3000 rpm at room temperature and were incubated for 24 h at 37 °C. Thereafter the 293vec-RD114 cells were trypsinized, transferred to gelatin-coated 10 cm dishes and expanded in 10 ml complete DMEM. Seventy two hours after transduction, the cells were harvested to determine their CAR expression by FACS (see below). Single, double and triple CAR expressing cells were enriched up to close to 100 % purity by positive selection using magnetic particles and surface antigen expression based method (EasySep) or FACS sorting (see below) and expanded in complete DMEM medium. At ∼ 90 % confluence, the viral vector containing supernatants of single, double or triple vector producing cells were harvested, spun down at 1800 rpm to remove any cells or debris and 10X concentrated using Retro-X (TakaraBio). Concentrated virus was snap-frozen by directly submerging it in liquid nitrogen and stored at −80 °C until use.

### 2.6 Magnetic-particle based cell separation of 293vec-RD114 stable virus-producer cell lines

For experiments illustrated in Fig. 2, Fig. 3, CAR2-LNGFR transduced 293vec-RD114 cells were sorted using the easysep APC positive selection kit II, following the manufacturer’s protocol after labeling the cells with LNGFR-APC (Biolegend). Similarly, CAR3-sII transduced cells were sorted using easysep FITC positive selection kit II after labeling cells with sII-FITC antibodies (THE™ NWSHPQFEK Tag Antibody [FITC], Genscript). Producer cells expressing both CAR2 and CAR3 were sequentially sorted. Sorted cells were plated in 10 cm dishes and expanded once ∼ 80 % confluency was reached.

### 2.7 Fluorescence-activated cell sorting of 293vec-RD114 stable virus-producer cell lines

For experiments illustrated in Fig. 4, Fig. 5, the FACSMelody Cell Sorter was used to simultaneously sort for PE-CF594 (dsRed, anti-CD38 CAR), PerCP-Cy5 (LNGFR, anti-TIM3 CAR) and FITC (sII, anti-CLEC12A CAR) positive cells. Conjugated mono-clonal antibodies used were: LNGFR-PerCP-Cy5 (Biolegend), sII-FITC (THE™ NWSHPQFEK Tag Antibody [FITC], Genscript).

### 2.8 Viral titer quantification

Viral titers were quantified using the Retro-X™ qRT-PCR Titration Kit (Takara Bio), following the manufacturer’s protocol. To compare SVP with DVP and TVP titers, the average of individual titers from SVP cells was calculated.

### 2.9 CAR T-cell transduction

Retroviral transduction of PHA-L stimulated T-cells with standard spinoculation/polybrene-based technique is described in detail previously.^12^ Briefly, after 48 h stimulation of 3x10^6^ PBMCs with lectin-like phytohemagglutinin (PHA-L, Sigma Aldrich) in culture medium (RPMI1640 + 10 % FBS + penicillin;100 U/ml, streptomycin; 100 μg/ml) at 37 °C, 5 % CO_2_, medium was removed until ∼ 1 ml was left and a total volume of 1 mL of concentrated virus was added to each well together with 4ug/mL polybrene. In case of double- or triple transductions, a volume-equalized mixture of two or three different virus batches were used (Table 2). The cells were spinoculated for 1 h at 3000 rpm at room temperature and further cultured at 37 °C, 5 % CO_2_. A second transduction was carried out after 24 h. Six hours later, the medium was replaced with complete culture medium supplemented with 50 IE/mL rhIL-2 (Proleukin®; Novartis). CAR transduction efficiency of T-cells was determined after 72 h, using flow cytometry-based detection of marker genes or CAR tags.

### 2.10 Bioluminescent imaging based cytotoxicity assays

Bioluminescence-based standard cytotoxicity assays were executed as described previously.^29^ Briefly, serial dilutions of mock or triple-transduced CAR T-cells were co-incubated with the luciferase-transduced malignant cell lines (20.000 cells per well in a 96-well plate) for 16–24 h. The luciferase signal produced by surviving malignant cells was then determined using a GloMax® 96 Microplate Luminometer (Promega) within 30 min of the addition of 125 μg/mL beetle luciferin (Promega). % lysis cells = (1 − (BLI signal in treated wells / BLI signal in untreated wells)) × 100 %.

## 3 Results

### 3.1 Generation of single, double or triple virus-producing 293vec-RD114 cells

We here propose a novel method to more conveniently and potentially more effectively transduce multiple transgenes into T-cells, by enabling the virus producer cell line (293vec-RD114 cells) to simultaneously produce a mixture of viruses carrying different genes of interest. To investigate the feasibility and applicability of our proposed method in the context of multi-CAR design, we used three retroviral vector plasmids encoding for distinct CAR transgenes, denoted as CAR1, CAR2, and CAR3, each co-expressing or labeled with a different marker (dsRed, LNGFR and sII, respectively)(Fig. 1A). To ensure stable and simultaneous production of double or triple viral vector mixes carrying CAR1, CAR2 and/or CAR3, we first transduced the 293vec-RD114 cells with volume-equalized mixtures of retroviral CAR1, CAR2 and CAR3 vectors, which were separately generated in Phoenix-Ampho cells (refer to Fig. 1 for a comprehensive visual representation of the methodology and Table 1 for all combinations). As controls, we carried out single transductions of 293vec-RD114 cells for every construct (Table 1 and Fig. 1B). As expected, and depending on the (combination) of virus used, transductions resulted in the expression of respective markers on 293vec-RD114 cells (Fig. 2A, B). 293vec-RD114 cells transduced with one, two or three CAR constructs were designated as single-virus-producing (SVP) cells, double-virus-producing (DVP) and triple-virus-producing (TVP) cells, respectively. We anticipated that a higher purity of virus producer cells could possibly lead to a higher viral titer production and higher subsequent purity in T-cells. For convenience, we used a selection method based on magnetic particles that bind to antibody complexes directed to specific cell surface antigens to select for CAR2 (LNGFR-APC) and/or CAR3 (sII-FITC) positive cells, to increase the extent of cells (co–)expressing the desired CARs in SVP, DVP and TVP cells. Both total LNGFR and sII positivity and double and triple positivity improved substantially upon a single round of sorting and individual CAR marker expression levels were equal or lower in DVP and TVP cells compared to SVP cells (Fig. 2C, D). The percentage of cells expressing CAR1 (dsRed) also increased in DVP and TVP cells, even though it was not individually selected for, probably because the fractions of CAR2 or CAR3 positive DVP and TVP cells expressed more CAR1 than the untransduced fractions.

### 3.2 Comparison of the efficiency of standard double and triple CAR T-cell transductions with CAR T-cell transduction with viruses produced by DVP and TVP cells

After generation DVP and TVP cells, we harvested and concentrated their viral supernatant to test their capacity to double and triple transduce T-cells. Volume equalized mixtures of virus batches from SVP cells were used as controls (Fig. 1C, Table 2). DVP and TVP derived viruses readily double and triple transduced T-cells from three independent donors to express the relevant CARs, indicating that they indeed produced a mixture of viruses carrying all transgenes (Fig. 3A). Furthermore, within each distinct combination, DVP- or TVP-derived viral mixtures led to equal or better total transduction efficiency of individual CARs compared to control SVP-derived viral particles (Fig. 3B) and a larger proportion of the desired double- or triple-positive transduced CAR T-cells in all three donors (Fig. 3C, supplementary Fig. 1). This was to some extent expected because of slightly higher viral titers produced by DVP and TVP cells compared to SVP-derived virus mixtures, however CAR1 + CAR3 CAR T-cells also had a larger fraction of double transduced cells, despite lower viral titers produced by DVP cells as compared to SVP cells for this combination (Supplementary Fig. 2).

### 3.3 TVP cells reproduce high triple CAR T-cell transduction rates

After demonstrating an optimized method for triple CAR T-cell generation, we used this proof-of-concept to make anti-CD38/CLEC12A/TIM3 (38/C/T) CAR T-cells. These CARs have all proven functional in a second generation CAR format (manuscripts in prep. and (29)). The three retroviral CAR vectors, with identical selection markers as the conceptual CAR1, CAR2 and CAR3, were transduced in 293vec-RD114 cells to create TVP cells. To achieve a higher purity, TVP cells were now sorted using fluorescence-activated cell sorting, resulting in a higher overall triple positive purity than before (Supplementary Figs. 3 and 4). We first assessed the reproducibility of TVP-based triple CAR T-cell transduction. We therefore harvested 3 distinct virus batches (B1-3) from the 38/C/T TVP cells, acquired at different days, and subsequently transduced primary human T-cells from three distinct donors (D1-3). Transduction led to high expression of all three CARs, as assessed by the expression of their surrogate markers (Fig. 4A). The percentage of T-cells expressing all three CARs was around 45 % for all three virus batches, and the proportion of cells lacking one or more CARs was stable as well (Fig. 4B). Notably, while inter-donor variability was clearly present, the inter-batch variability was negligible, illustrating the reproducibility of this method (Fig. 4C, Supplementary Fig. 5).

### 3.4 Triple anti-CD38/CLEC12a/TIM3 CAR T-cells generated with TVP cells are functionally active

Finally we sought to test the functional activity of the 38/C/T CAR T-cells. We therefore selected a CD38-postive cell line UM9, performed CRISPR/Cas9-mediated CD38 knockout and ectopically expressed either TIM3 or CLEC12A, to generate four cell lines each positive for only one of the target antigens to be able to individually assess the activity of all CARs (Fig. 5A). The 38/C/T CAR T-cells induced significantly more lysis than control untransduced T-cells in the cell lines with either CD38, TIM3 or CLEC12A expression, but not in the cell line without target antigen expression (Fig. 5B). Conclusively, this demonstrates the functional activity of each individual CAR and therefore the successful application of TVP cells to generate triple CAR T-cells.

## 4 Discussion

Despite the enormous success of cell therapies such as CAR T-cell therapy, it also becomes increasingly clear that not all cancers will entirely be eradicated using conventional “simple” CAR T-cell therapies and that additional engineering is required for improving effectivity, persistence and safety. Since the expression of multiple transgenes in human T-cells can be challenging and may accompany low expression levels or high inter-experiment variability, we sought a convenient method that is efficient and reproducible. We show that virus from DVP and TVP cells substantially improves success in these measures.

We believe that research using advanced T-cell engineering can benefit from this method. Using our method for the generation of clinical grade virus batches may however require additional investigation. One important potential drawback is the multiple gene integrations in the host genome, which may increase the risk of oncogenic transformation.^30^ Although secondary malignancies are still extremely rare and are heavily outweighed by the chance of therapy success,^31^, ^32^ the potential oncogenic impact of higher number of γ-retrovirus integrations is a general concern and needs to further investigated before clinical implementation of this methodology.

Sorting of TVP cells will likely always be required to obtain a high purity of triple CAR^+^ T-cells and this may seem to reduce the convenience of this method. However, sorting, or selection of a high titer-producing clone of stable virus-producer cell lines is also preferred with conventional SVP cell lines and in our method only one TVP needs to be sorted, instead of three individual SVP cell lines. More importantly, CAR + T-cells likely do not need to be sorted as the purity is already very high as demonstrated in Fig. 4. This is a major advantage, as CAR T-cell sorting is more problematic because this would need to be done every round of CAR T-cell production. TVP cell line sorting needs to be performed only once and virus production produced in separate batches leads to very stable transduction efficiencies, as shown in Fig. 4B and D.

The improved overall transduction efficiency and reduction of mixed populations when using DVP and TVP cells could not be completely explained by improved viral titers (Supplementary Fig. 1). One possible explanation is that γ-retroviruses co-package two strands of RNA.^33^ For example, in the case of DVP or TVP cells, one viral particle could harbor one copy of CAR1 RNA and one copy of CAR2 RNA. Endocytosis of this viral particle can therefore simultaneously lead to integration of CAR1 and CAR2 in the host genome. In contrast, in SVP cells, even when viruses from different SVP cells are combined, they always harbor a duplicate of the same RNA strand. While this co-packaging may potentially explain the improved efficiency of this method, it also brings up new questions about potential recombination of the two RNA strands. The implications of such theoretical recombination varies depending on the chosen strategy, and may be minimal when, for example, “OR-gate” CAR T-cells are used which are receptors that are functionally very similar, but could have more impact when recombination could lead to unwanted products. More research will be needed to elucidate the consequence and impact of RNA co-packaging in DVP and TVP cells.

In our study we successfully incorporated up to three CARs in T-cells to show one of the potential application of this method. Tri-specific CAR T-cells, as demonstrated here, have broader reactivity and may prove useful to prevent relapse after CAR T-cell therapy due to antigen-loss, a considerable challenge in clinically available CAR T-cell products or malignancies with wide-ranging tumor heterogeneity.^34^, ^35^, ^36^, ^37^ The methodology can also be readily used to develop and rapidly test dual CAR T-cell strategies based on “AND” gating to improve the tumor cell specificity. For example, we have recently demonstrated the effectivity and safety of CD138/CD38 “AND-gate” and BCMA/CD38 “OR gate” dual CAR T cells.^12^, ^13^ If necessary, a third (stimulatory or costimulatory) CAR could be readily incorporated with our method, to further improve the specificity and reduce the chance of antigen escape.

Understandably our method will also allow the expression of transgenes other than CARs in T-cells. Recently multiple studies have shown that the incorporation, knock-out or targeting of certain genes can improve the metabolic fitness, memory phenotype and/or persistence of CAR T-cells.^14^, ^38^, ^39^, ^40^, ^41^, ^42^, ^43^, ^44^, ^45^, ^46^, ^47^, ^48^, ^49^ Applying our method, it may be easier to induce expression of one or two of such genes besides simultaneously expressing one or two synthetic receptors such as CARs.

## CRediT authorship contribution statement

**Jort J van der Schans:** Conceptualization, Data curation, Visualization, Writing – original draft. **Afroditi Katsarou:** Investigation. **George Kladis:** Investigation. **Citlali Bar:** Investigation. **Max Medina Ramirez:** Supervision, Methodology. **Maria Themeli:** Writing – review & editing, Resources. **Tuna Mutis:** Writing – review & editing, Supervision, Funding acquisition.

## Declaration of competing interest

The authors declare the following financial interests/personal relationships which may be considered as potential competing interests: Tuna Mutis reports financial support was provided by Amsterdam UMC Location VUmc. Jort van der Schans reports a relationship with Bastion Therapeutics that includes: consulting or advisory. Tuna Mutis reports a relationship with Dutch Cancer Society that includes: funding grants. Maria Themeli reports a relationship with Sangamo Therapeutics Inc that includes: consulting or advisory. Maria Themeli has patent #WO2014165707 with royalties paid to Memorial Sloan Kettering Cancer Center. Maria Themeli has patent #WO2022204129 pending to Memorial Sloan Kettering Cancer Center, Amsterdam UMC. If there are other authors, they declare that they have no known competing financial interests or personal relationships that could have appeared to influence the work reported in this paper.
