# Supplementary material for: A convenient viral transduction based method for advanced multi-engineering of primary human (CAR) T-cells
Source: J Genet Eng Biotechnol. 2024 Nov 28;22(4):100446. doi: 10.1016/j.jgeb.2024.100446 (PMC11629549; doi:10.1016/j.jgeb.2024.100446)
Supplement: Supplementary Data 1 [file mmc1.pptx]

## Slide 1
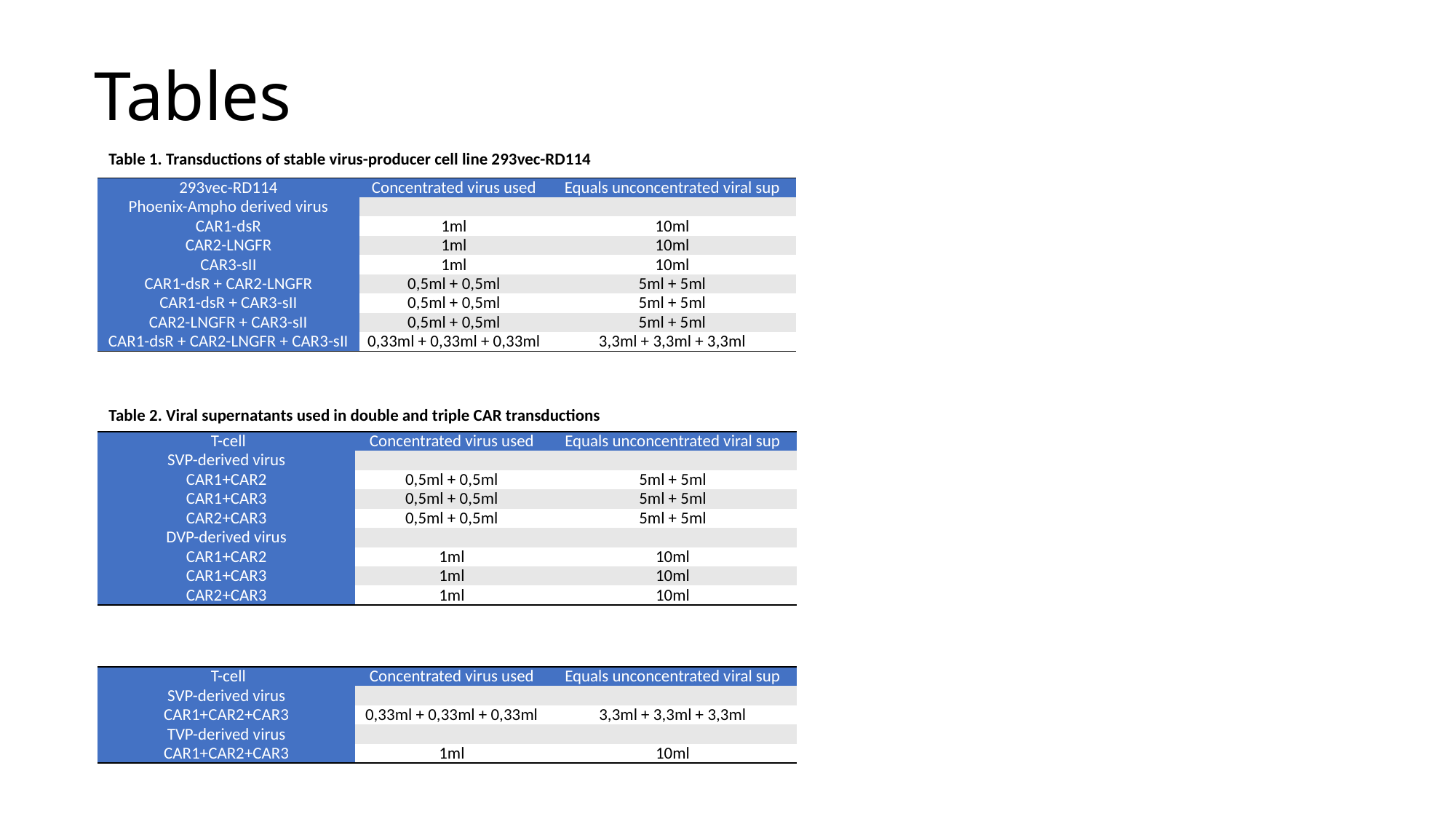

# Tables
Table 1. Transductions of stable virus-producer cell line 293vec-RD114
| 293vec-RD114 | Concentrated virus used | Equals unconcentrated viral sup |
| --- | --- | --- |
| Phoenix-Ampho derived virus | | |
| CAR1-dsR | 1ml | 10ml |
| CAR2-LNGFR | 1ml | 10ml |
| CAR3-sII | 1ml | 10ml |
| CAR1-dsR + CAR2-LNGFR | 0,5ml + 0,5ml | 5ml + 5ml |
| CAR1-dsR + CAR3-sII | 0,5ml + 0,5ml | 5ml + 5ml |
| CAR2-LNGFR + CAR3-sII | 0,5ml + 0,5ml | 5ml + 5ml |
| CAR1-dsR + CAR2-LNGFR + CAR3-sII | 0,33ml + 0,33ml + 0,33ml | 3,3ml + 3,3ml + 3,3ml |
Table 2. Viral supernatants used in double and triple CAR transductions
| T-cell | Concentrated virus used | Equals unconcentrated viral sup |
| --- | --- | --- |
| SVP-derived virus | | |
| CAR1+CAR2 | 0,5ml + 0,5ml | 5ml + 5ml |
| CAR1+CAR3 | 0,5ml + 0,5ml | 5ml + 5ml |
| CAR2+CAR3 | 0,5ml + 0,5ml | 5ml + 5ml |
| DVP-derived virus | | |
| CAR1+CAR2 | 1ml | 10ml |
| CAR1+CAR3 | 1ml | 10ml |
| CAR2+CAR3 | 1ml | 10ml |
| T-cell | Concentrated virus used | Equals unconcentrated viral sup |
| --- | --- | --- |
| SVP-derived virus | | |
| CAR1+CAR2+CAR3 | 0,33ml + 0,33ml + 0,33ml | 3,3ml + 3,3ml + 3,3ml |
| TVP-derived virus | | |
| CAR1+CAR2+CAR3 | 1ml | 10ml |

## Slide 2
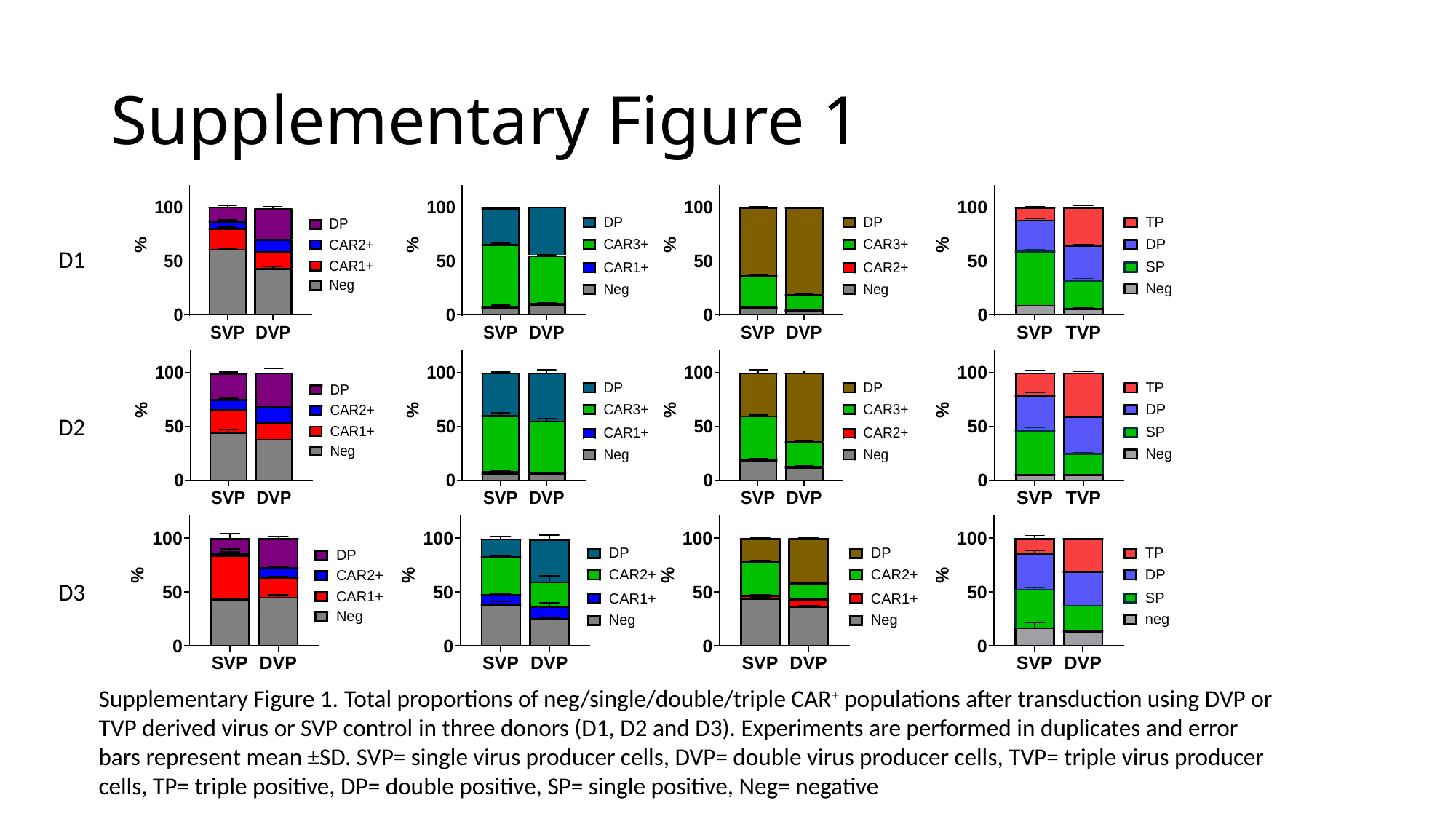

# Supplementary Figure 1
D1
D2
D3
Supplementary Figure 1. Total proportions of neg/single/double/triple CAR+ populations after transduction using DVP or TVP derived virus or SVP control in three donors (D1, D2 and D3). Experiments are performed in duplicates and error bars represent mean ±SD. SVP= single virus producer cells, DVP= double virus producer cells, TVP= triple virus producer cells, TP= triple positive, DP= double positive, SP= single positive, Neg= negative

## Slide 3
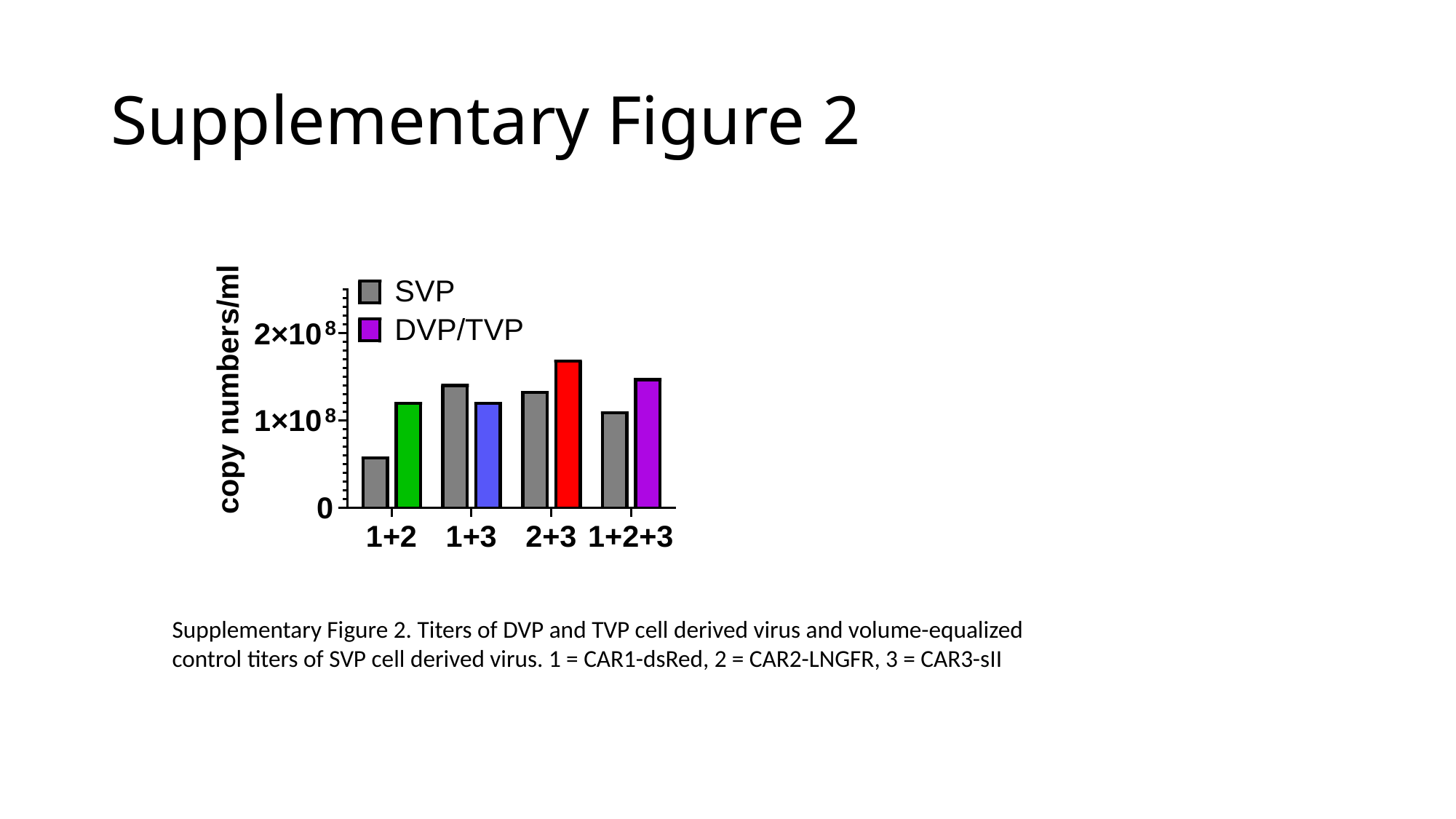

# Supplementary Figure 2
Supplementary Figure 2. Titers of DVP and TVP cell derived virus and volume-equalized control titers of SVP cell derived virus. 1 = CAR1-dsRed, 2 = CAR2-LNGFR, 3 = CAR3-sII

## Slide 4
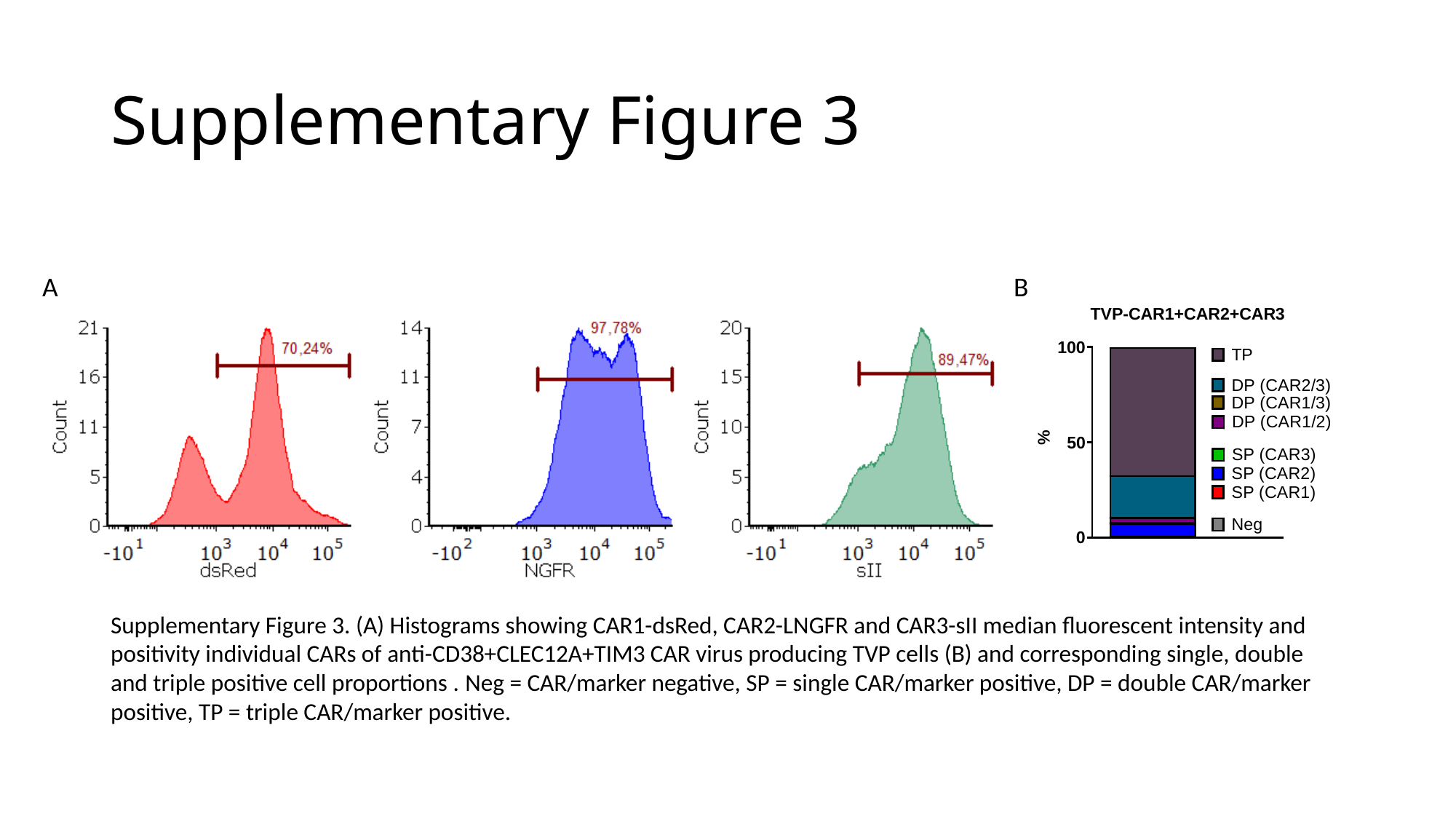

# Supplementary Figure 3
A
B
Supplementary Figure 3. (A) Histograms showing CAR1-dsRed, CAR2-LNGFR and CAR3-sII median fluorescent intensity and positivity individual CARs of anti-CD38+CLEC12A+TIM3 CAR virus producing TVP cells (B) and corresponding single, double and triple positive cell proportions . Neg = CAR/marker negative, SP = single CAR/marker positive, DP = double CAR/marker positive, TP = triple CAR/marker positive.

## Slide 5
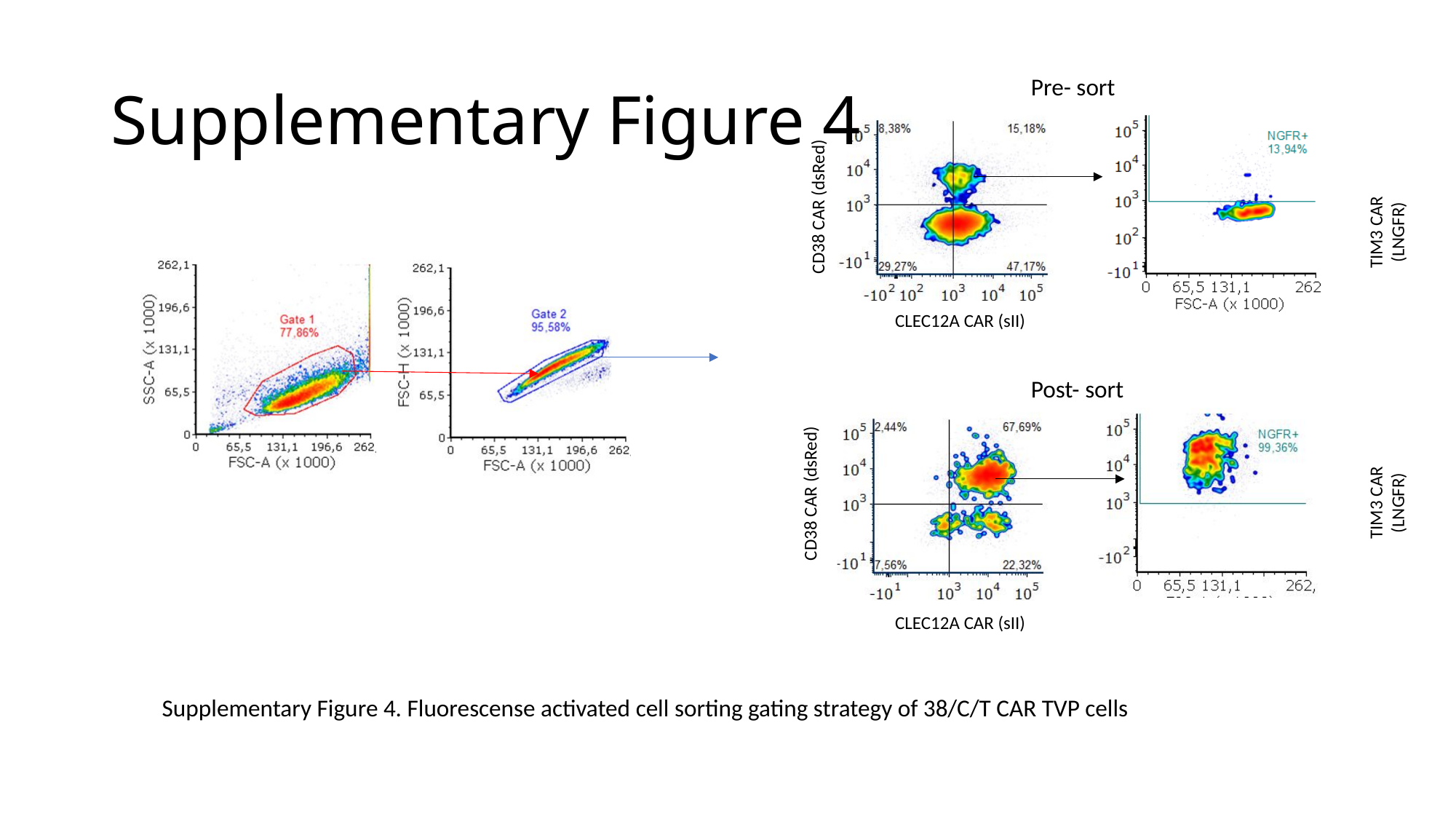

# Supplementary Figure 4
Pre- sort
CD38 CAR (dsRed)
TIM3 CAR (LNGFR)
CLEC12A CAR (sII)
Post- sort
TIM3 CAR (LNGFR)
CD38 CAR (dsRed)
CLEC12A CAR (sII)
Supplementary Figure 4. Fluorescense activated cell sorting gating strategy of 38/C/T CAR TVP cells

## Slide 6
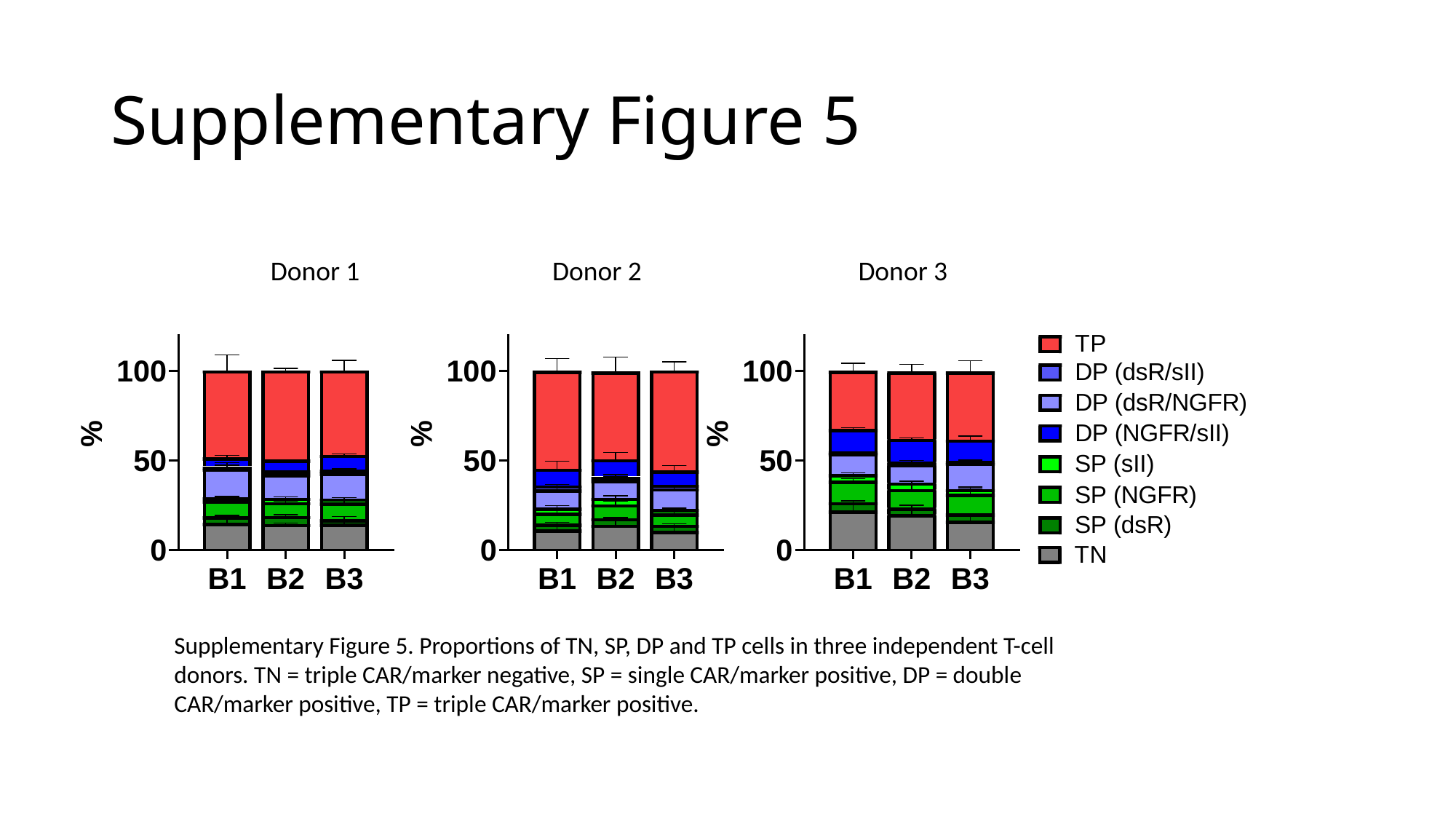

# Supplementary Figure 5
Donor 1
Donor 2
Donor 3
Supplementary Figure 5. Proportions of TN, SP, DP and TP cells in three independent T-cell donors. TN = triple CAR/marker negative, SP = single CAR/marker positive, DP = double CAR/marker positive, TP = triple CAR/marker positive.
